# Volatile‐mediated antagonism of soil bacterial communities against fungi

**DOI:** 10.1111/1462-2920.14808

**Published:** 2019-11-04

**Authors:** Xiaogang Li, Paolina Garbeva, Xiaojiao Liu, Paulien J. A. klein Gunnewiek, Anna Clocchiatti, Maria P. J. Hundscheid, Xingxiang Wang, Wietse de Boer

**Affiliations:** ^1^ Co‐Innovation Center for Sustainable Forestry in Southern China, College of Biology and the Environment Nanjing Forestry University Nanjing 210037 China; ^2^ Department of Microbial Ecology Netherlands Institute of Ecology NIOO‐KNAW Wageningen 6708 PB The Netherlands; ^3^ CAS Key Laboratory of Soil Environment and Pollution Remediation Institute of Soil Science, Chinese Academy of Sciences Nanjing 210008 China; ^4^ College of Plant Protection Southwest University Chongqing 400715 China; ^5^ Soil Biology Group Wageningen University Wageningen 6708 PB The Netherlands

## Abstract

Competition is a major type of interaction between fungi and bacteria in soil and is also an important factor in suppression of plant diseases caused by soil‐borne fungal pathogens. There is increasing attention for the possible role of volatiles in competitive interactions between bacteria and fungi. However, knowledge on the actual role of bacterial volatiles in interactions with fungi within soil microbial communities is lacking. Here, we examined colonization of sterile agricultural soils by fungi and bacteria from non‐sterile soil inoculums during exposure to volatiles emitted by soil‐derived bacterial communities. We found that colonization of soil by fungi was negatively affected by exposure to volatiles emitted by bacterial communities whereas that of bacteria was barely changed. Furthermore, there were strong effects of bacterial community volatiles on the assembly of fungal soil colonizers. Identification of volatile composition produced by bacterial communities revealed several compounds with known fungistatic activity. Our results are the first to reveal a collective volatile‐mediated antagonism of soil bacteria against fungi. Given the better exploration abilities of filamentous fungi in unsaturated soils, this may be an important strategy for bacteria to defend occupied nutrient patches against invading fungi. Another implication of our research is that bacterial volatiles in soil atmospheres can have a major contribution to soil fungistasis.

## Introduction

Fungi and bacteria are two main groups of microorganisms involved in the decomposition of organic compounds in soils (Rousk and Frey, 2015; de Menezes *et al*., 2017). These compounds range from simple sugars in root exudates to highly recalcitrant lignin‐rich materials in plant cell walls (Kuzyakov, 2010). Despite general differences between fungi and bacteria in their abilities to access and decompose labile and recalcitrant compounds, competition for organic energy sources appears to be the major type of interaction in soils (de Boer *et al*., 2005; Hannula *et al*., 2017). An important strategy in competition for energy sources is the production of inhibitors often referred to as interference competition or antagonism (Hibbing *et al*., 2010; Hennessy *et al*., 2017). The chemical and physical properties of inhibitors determine the mode of action and the spectrum of activity against organisms can vary from broad to specific (Raaijmakers and Mazzola, 2012; Deveau *et al*., 2018). A group of inhibitors that are of particular interest for antagonistic microbial interactions in soils are volatile compounds. Most soils are not saturated with water implying that part of the pores is air‐filled. The production of inhibiting compounds diffusing as volatiles through air‐filled pores increases the distance over which antagonistic interactions can take place (Insam and Seewald, 2010; Effmert *et al*., 2012).

Air‐filled pores limit the dispersal possibilities of unicellular bacteria whereas hyphal growth enables filamentous fungi to cross those (Pajor *et al*., 2010). Interestingly, there are bacterial species that take advantage of ‘fungal bridges’ by moving in water‐films along fungal hyphae (Nazir *et al*., 2010). However, a general picture arising from this difference in dispersal abilities between bacteria and fungi is that unicellular bacteria are mostly restricted to the energy sources in local spots whereas hyphal‐forming fungi have the ability to get access to these local spots (Boswell *et al*., 2007; Wolf *et al*., 2013). In such a scenario, it could pay off for bacteria to collectively produce fungus‐inhibiting volatiles to reduce the possibility for fungi to invade local organic patches occupied by bacteria. Therefore, we hypothesized that long distance volatile‐mediated antagonism is especially of importance in soils for competition of bacteria against fungi.

Current knowledge on inhibiting activities of bacterial volatiles is mainly based on two‐compartment studies using semi‐solid media with a bacterial volatile producer in one compartment and a fungal receiver in the other. Such studies have revealed that many fungal receivers are restricted in their growth when exposed to bacterial volatiles, but that the medium composition is strongly influencing the results (Effmert *et al*., 2012; Schmidt *et al*., 2015). Hol *et al*. (2015) increased the complexity of two‐compartment studies by examining the effect of bacterial diversity on the intensity of fungal pathogen suppression by volatiles. Several volatiles with known antifungal activity were only produced by the most species‐rich soil bacterial communities, but effects of volatiles on bacteria were not examined. In general, little attention has been paid to the effects of bacterial volatiles on other bacteria. However, a recent study reporting on pairwise volatile interactions indicated that bacteria are less sensitive to bacterial volatiles than fungi (Ossowicki *et al*., 2017). Yet, it is not clear if predominant antifungal effects are occurring with volatiles produced by soil bacterial communities, as the antagonistic effects found in pairwise interactions are often not representative for those found in communities (de Boer, 2017).

The aim of the current study was to examine antifungal and antibacterial activity of volatiles produced by soil bacterial communities. To this end, we exposed soil microbial communities colonizing sterile agricultural soils to volatiles emitted by bacterial communities originating from those soils. Growth and composition of fungi and bacteria colonizing sterile agricultural soils were determined. Our results revealed that bacterial community volatiles predominantly suppressed the development of fungal biomass and affected fungal community composition whereas development of bacteria was hardly affected.

## Materials and methods

### *Soil characteristics*

Soil samples were collected to a depth of around 15 cm from three agricultural fields in the Netherlands in January 2017. Soils were sieved (4 mm mesh size) and stored in plastic bags at 4 °C until further use. These soils were characterized as sandy soils with different pH values (5.1, 6.6 and 7.6). Other physico‐chemical properties are given in Table S1. The fresh soil was sterilized twice at 121 °C for 3 h with an overnight interval. Sterility was confirmed by spreading 0.5 g of the sterilized soil onto tryptic soy broth (TSB) agar and potato dextrose agar media (composition of media in Table S2) and incubation for 6 days at 20 °C.

### *Testing impact of bacterial community volatiles on microbial community development in sterile soils*

**Figure 1:**
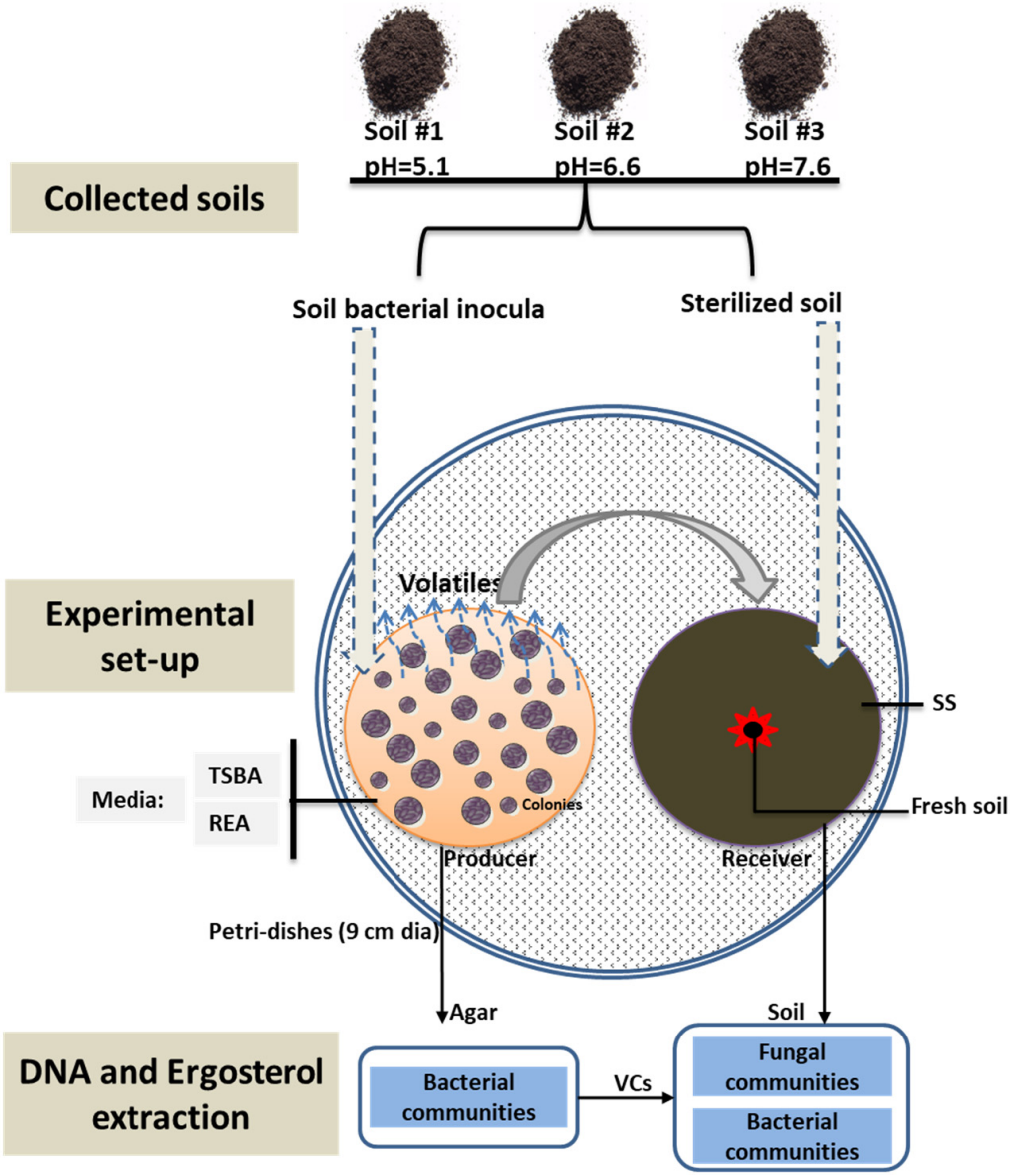
Flow diagram of the key experimental procedure in this study. The sandy soils #1, #2, and #3 were collected from three agricultural fields in the Netherlands. The left small Petri‐dish served as bacterial community volatile‐producing compartment. Two growth media, namely REA and 1/10 TSB agar (TSBA) containing the fungal inhibitors cycloheximide and thiabendazole, were used to grow bacteria extracted from the three soils. The other small Petri‐dish containing 20 g sterilized soil was used as the volatiles receiving compartment. After closing the big Petri‐dish (9 cm dia) and pre‐exposure for 3 days to volatiles originating from the producer compartment, 0.5 g of fresh soil was added to the centre of the receiver compartment. Next, the big Petri‐dish was closed again and development of fungal and bacterial biomass and communities was determined after 14 days of incubation. As controls, producer compartments (*n* = 18) containing the respective growth media without bacteria inoculums were used.

The effect of bacterial community volatiles on microbial development was studied using large Petri‐dishes (9 cm dia.) containing two small Petri‐dishes (3.5 cm dia.). A flow diagram of the key steps in this study is shown in Fig. 1. One of the small Petri‐dishes (volatile receiving compartment) was used to examine soil microbial community developments during exposure to bacterial volatiles and was filled with 15 g sterilized agricultural soil (60% WHC); the other (volatile‐producing compartment) was used for the emission of volatiles by bacterial communities and contained agar media [root‐exudate agar (REA) or 1/10 strength TSB agar (1/10 TSBA)]. The composition of both agar media is given in Table S2. Both agar media in the volatile‐producing compartment contained the fungal inhibitors cycloheximide (Sigma‐Aldrich, St. Louis, MO) and thiabendazole (Sigma‐Aldrich) (Table S2). The combination of these inhibitors was found to be effective to prevent fungal growth in previous experiments (Hol *et al*., 2015).

Bacterial inoculums for the volatile‐producing compartments were obtained from the three agricultural soils as described by Hol *et al*. (2015). Briefly, portions of soil equivalent to 5 g dry weight and 50 ml phosphate buffer (KH_2_PO_4_, 1 g/l, pH 6.5) were mixed on a rotary shaker for 1.5 h at 4 °C, followed by 1 min sonification at 47 kHz twice and shaking for 0.5 h. Next, the suspensions were filtrated through a 5‐μm filter to remove a large proportion of fungal propagules. From the filtered soil suspensions 50 μl was plated on the agar media. Plating with 50 μl of autoclaved phosphate buffer (1 g/l) served as control. After sealing the big Petri‐dishes pre‐incubation was at 20 °C for 3 days with the aim to have the sterilized soil in the volatiles receiving compartment already exposed to volatiles produced by the developing bacteria in the volatile‐producing compartments. Next, the big Petri‐dishes were opened and 0.5 g fresh soil of each of the three agricultural soils was added to the centre of the volatiles receiving compartments containing the corresponding sterile soil (Fig. 1). The big Petri‐dishes were closed again and incubated further at 20 °C for 14 days.

In total, there were *n* = 36 units (3 soils × 2 media × 3 replicates for production of volatiles by bacterial communities and controls respectively). After incubation for 2 weeks, soil samples from the volatile receiving compartments were collected and homogenized, and part of these samples were taken to determine the fungal biomass. The rest of the soil samples were stored at −20 °C until extraction of DNA. Bacterial colonies growing on the two agar media in the volatile‐producing compartments were suspended in sterile water (2 times in 3 ml sterile water), and then stored at −20 °C for DNA extraction.

### *Determination of fungal and bacterial biomass in volatiles‐exposed soils*

The amount of ergosterol, a sterol occurring in fungal membranes, was determined to quantify fungal growth in the sterile soil in the volatile receiving compartments (De Ridder‐Duine *et al*., 2007). Bacterial abundance in all soil samples was determined by quantitative PCR (qPCR) using the Eub 338 and Eub 518 primer set for the bacterial 16S rRNA gene (Muyzer *et al*., 1993). Total genomic DNA was extracted from 0.35 g of the subsamples of the inoculated sterilized soil (volatile receiving compartment) using a MoBio Power Soil Extraction kit according to the supplier's instructions (MoBio Laboratories, Carlsbad, CA). Each 25 μl reaction mixture consisted of 12.5 μl of Sybr green mix (Bioline, GC‐Biotech) with 4 mg/ml bovine serum albumin, 5 μM each primer, and 5 μl of template DNA with 10‐fold dilution. The standard curves were generated using a 10‐fold dilution series of a plasmid containing a full‐length copy of the 16S rRNA gene of *Collimonas fungivorans*. PCRs were run on a Rotor‐Gene 3000 (Qiagen, Hilden, Germany) and started with 15 min at 95 °C, followed by 40 amplification cycles each of 95 °C for 60 s, 53 °C 50 s, and 72 °C 60 s. Bacterial gene copy numbers were generated using a regression equation for each assay relating the cycle threshold (*C*_*t*_) value to the known number of copies in the standards. All qPCR reactions were run in quadruplicate with DNA extracted from each soil sample.

### *Analysis of fungal and bacterial communities*

Characterization of bacterial and fungal communities colonizing the sterile soils in the volatile receiving compartments was performed using the DNA extracted for qPCR analyses. In addition, community composition analysis was performed for the bacteria that developed on the two agar media in the volatile‐producing compartments using DNA extracted from the agar plates. To this end, bacterial suspensions collected from the agar surface (see above) were first centrifuged for 10 min at 13 000 rpm after which the pellets were used for DNA extraction with the Mobio Power Soil DNA Isolation kit (Qiagen, Valencia, CA) according to the manufacturer's instructions. Amplicon libraries were prepared for all 36 soil samples of the receiver compartments and for triplicate samples of the three soil bacterial communities on the two agar media in the producer compartments (total 18 samples). This was done using tagged bacterial and fungal universal primers (F515/R806 for bacteria; ITS9/ITS4 for fungi), which target the V4 region of the 16S rRNA gene and the internal transcribed spacers 1 respectively (Caporaso *et al*., 2010). For the producer compartments, amplicon libraries were only prepared for bacteria. More information on thermocycling conditions and purification of samples is given by Li *et al*. (2018). Amplicons from three reactions per sample were combined and cleaned with the Qiagen PCR purification kit (Qiagen). The 16S rRNA and ITS1 genes in the pooled samples were sequenced on an Illumina MiSeq platform at BIG Genomics (Shenzhen, China) using paired‐end reads. All MiSeq data were uploaded to the European Nucleotide Archive (https://www.ebi.ac.uk/ena/) under the study accession number PRJEB34251.

The analysis was performed by the Hydra pipeline version 1.3.3 implemented in Snakemake (Köster and Rahmann, 2012). Reads were first quality filtered, followed by trimming adapters sequences and removing PhiX contaminants, using BBDuk2 from the BBMap tool suite (Bushnell, 2015). The fastq‐mergepairs command of VSEARCH (Rognes *et al*., 2016) was used to merge paired‐end reads. Sequences were converted to FASTA format and concatenated into a single file. All reads are clustered into OTUs using the UPARSE strategy by dereplication, sorting by abundance with at least two sequences and clustering using the UCLUST smallmem algorithm (Edgar, 2010). These steps were performed with VSEARCH. Next, chimeric sequences were detected using the UCHIME algorithm in de novo mode (Edgar *et al*., 2011) implemented in VSEARCH. All reads before the dereplication step were mapped to OTUs using the USEARCH_global method implemented in VSEARCH to create an OTU table and converted to BIOM‐Format (McDonald *et al*., 2012). Finally, taxonomic information for each OTU was added to the BIOM file by aligning the sequences to the SILVA database (release 128) (Quast *et al*., 2013) using SINA for bacteria and running the RDP Classifier re‐trained on the UNITE database release 7.2 (Kõljalg *et al*., 2013) for fungi.

### *Trapping and analysis of bacterial volatiles*

To examine whether the bacterial community origin affected the volatile profiles produced on agar, glass Petri‐dishes with ‘chimney’ lids were used, in which steel traps with 150 mg Tenax TA and 150 mg Carbopack B (Markes International, Llantrisant, UK) were fixed (Garbeva *et al*., 2014a). Collection of bacterial volatiles was done for bacterial communities growing on REA as this growth medium resembles the situation in soil more than TSB agar. Three independent replicates were used for each soil type. Sterile Petri‐dishes containing REA without bacterial inoculation served as background controls. Volatiles were collected for 24 h after 7 days of incubation. Next, the traps were removed, capped, and stored at 4 °C until analysis using GC‐Q‐TOF QTOF (model Agilent 7890B GC and the Agilent 7200A QTOF, Santa Clara, CA). Detailed information on volatile analysis is given by Hol *et al*. (2015). For an overall representation of volatile profiles of the bacterial communities, a partial least square‐discriminant analysis was made based on peak areas with 95% confidence regions as described in Tyc *et al*. (2015). Identification of metabolites was performed using NIST‐MS Search and accurate mass, retention indices, and spectra match factor using NIST 2014 (National Institute of Standards and Technology, http://www.nist.gov). The linear retention indexes (lri) values were compared with those found in the NIST and the NIOO lri database. Some identified compounds were also verified by comparing mass spectra and lri values of pure compounds.

### *Statistical analyses*

Analysis of variance (ANOVA) and Fisher's *t* test were used to test the effects of exposure to bacterial community volatiles on fungal (ergosterol) and bacterial (qPCR) biomass using SPSS (v. 19). The ratio of fungi to bacteria was calculated using the formula: *F*/*B* = fungal biomass/bacterial biomass. Fungal biomass (*F*) was calculated according to: *F* = ∑*E* × *F*, where *E* is the amount of ergosterol determined, and where *F* = 182 was used as conversion factor from ergosterol to biomass (Gessner and Chauvet, 1993). Bacterial biomass (*B*) was calculated based on bacterial copy numbers referring to the method of Ingraham and Neidhardt (1987).

Principal coordinates analysis plots were used to visualize the community structure among samples, and were generated from the Bray–Curtis similarity index matrices of all samples and created using the PAST software program (Hammer *et al*., 2001). Differences in the relative abundance of the fungal and bacterial phylotypes between treatments were compared using STAMP (Version 8.30) with the gplots package and differences were considered to be significant at *p* < 0.05 (Welch's *t*‐test). The confidence interval was estimated using the Newcombe–Wilson method. The individual effects of the three soil origins and volatile treatments (bacteria producing volatiles on ARE and 1/10 TSBA) on the composition of fungal genera and bacterial families were quantified using two‐way PERMANOVA in R packages vegan. In addition, one‐way PERMANOVA analysis was performed to test the effects of soil origins on the composition of bacteria growing on the agar media in the volatile‐producing compartments.

## Results

### *Responses of microbial growth in soil to bacterial community volatiles*

**Figure 2:**
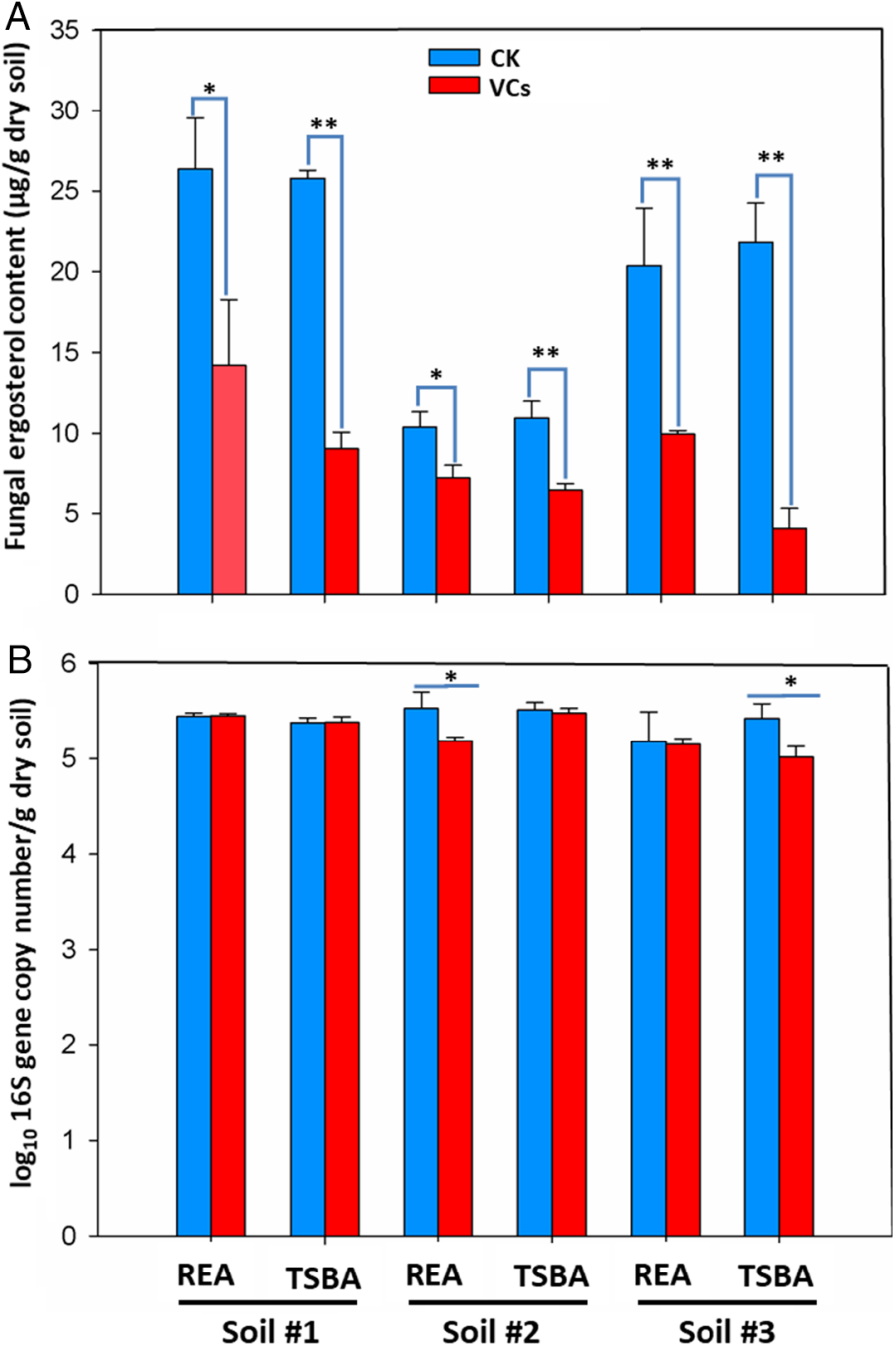
Effect of volatiles produced by soil bacterial communities growing on two agar media on fungal biomass (A), and bacterial 16S gene copy numbers (B) developing in sterile soil receiver compartments. Colonization of sterile soil by fungi and bacteria in volatile receiving compartments started with addition of a small amount of non‐sterile soil and growth was determined after 14 days of exposure to volatiles produced by bacteria growing on/in ‘REA’: root‐exudate agar, and ‘TSBA’: 1/10 TSB agar. Controls consisted of the same growth media without inoculation of bacteria. Mean values and standard deviations are presented (*n* = 3). The asterisks indicate that differences between soils exposed to bacterial volatiles and controls are statistically significant (*p < 0.05, **p < 0.01) as determined by Fisher's LSD test.

The amount of fungal biomass (ergosterol content) in sterile agricultural soils inoculated with a small amount of the same non‐sterile soils was affected by exposure to volatiles produced by soil bacterial communities. For all three sterile soils exposure to volatiles emitted by bacterial communities resulted in strong (34.5%–81.5%) reduction of fungal biomass production (Fig. 2A, Table S3, overall, *P* < 0.001). The extent of inhibition of fungal biomass production by volatiles was affected by the growth conditions of the bacteria in the volatile‐producing compartment and was highest for 1/10 TSBA (Table S4). On the contrary, bacterial colonization of sterile soils exposed to bacterial community volatiles was not significantly or only very limited (0.5%–7.5%) affected (Fig. 2B, Table S5, overall, *P* > 0.05). Consequently, fungal/bacterial ratios in colonized sterile soils exposed to bacterial community volatiles were strongly reduced (Fig. S1, overall, *P* < 0.05).

### *Microbial community composition response to bacterial community volatiles*

**Figure 3:**
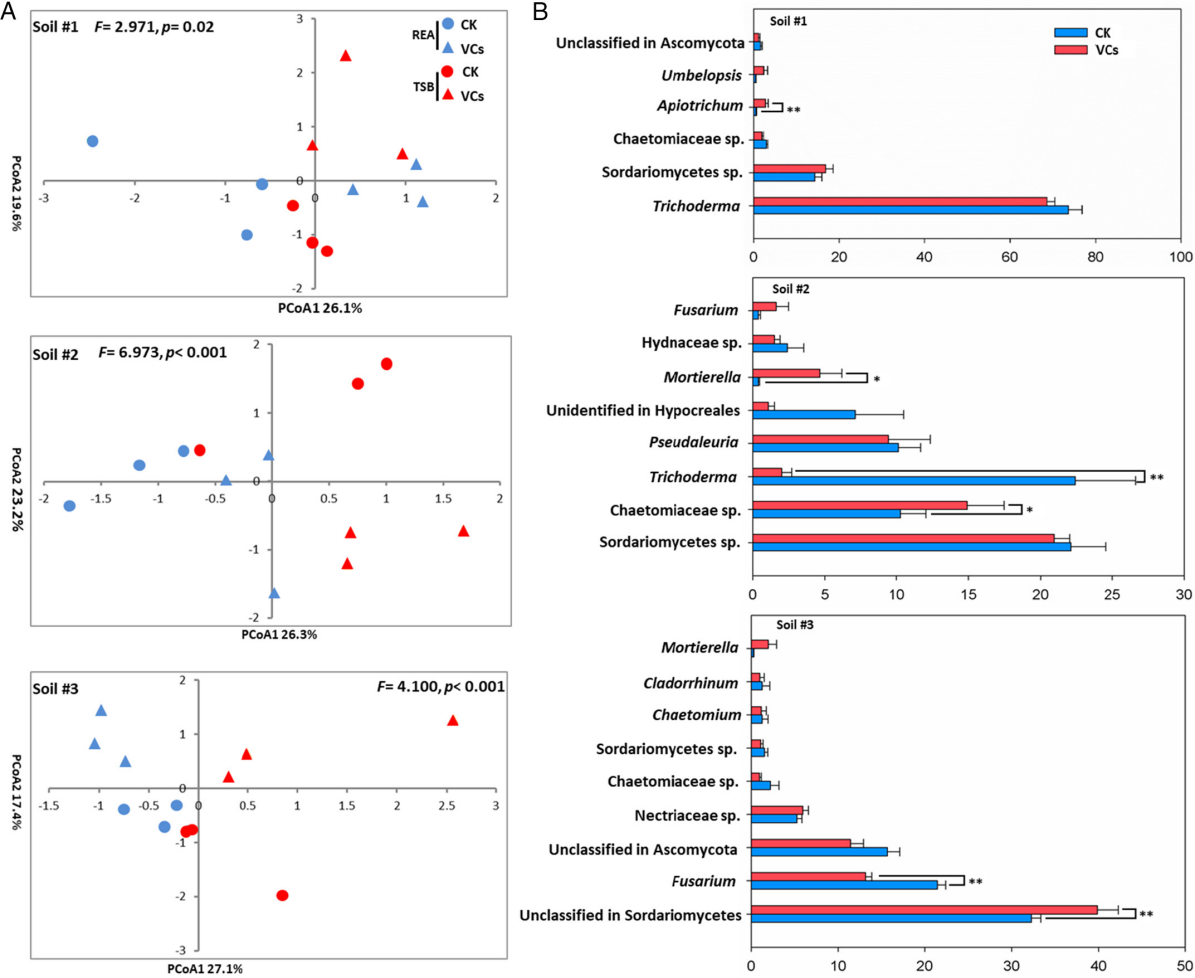
A. Principal component analysis based on Bray–Curtis results of the composition of fungal communities in receiver compartments (sterile soils inoculated with fresh soil) that were exposed (VCs) or not (CK) to volatiles produced by soil bacterial communities growing on two agar media (REA, 1/10 TSBA). The percentages on the axis labels represent the percentages of variation explained by the principal coordinates. B. Mean proportion (*n* = 6) of the most abundant fungal genera (ITS sequences >1%) in soils that were exposed (VCs) or not (CK) to volatiles produced by soil bacterial communities growing on two agar media (REA, 1/10 TSBA). *p < 0.05, **p < 0.01.

After filtering based on basal quality control, a total of 865,521 bacterial and 473,026 fungal sequence reads were obtained for the 36 soils samples in the receiver compartments. An average of 24,042 (range: 5701–40,882) bacterial sequences and 13,141 (range: 4081–26,132) fungal sequences were grouped into 4,235 bacterial OTUs and 447 fungal OTUs respectively. To examine the effect of bacterial community volatiles on microbial community structure in sterile soils inoculated with fresh soil, Bray–Curtis similarity matrices were constructed based on relative abundances of fungal and bacterial OTUs. The effect of soil origin on fungal community composition was more pronounced than that of exposure to volatiles (Table S6, *F* = 81.19, *p* < 0.001). Yet, PERMANOVA analysis for each of the three soils showed that exposure to bacterial community volatiles significantly influenced the composition of fungal colonizers (Fig. 3A). These effects were strongest for the neutral soil (*F* = 6.973, *p* < 0.001), followed by the alkaline soil (*F* = 4.100, *p* = 0.004), and were least for the acid soil (*F* = 2.971, *p* = 0.02) (Table S6). By contrast, the composition of bacterial communities colonizing sterile soils during exposure to bacterial volatiles showed no overall significant differences with the controls (soil #1: *F* = 1.878, *p* = 0.151; soil #2: *F* = 2.621, *p* = 0.069; soil #3: *F* = 1.882, *p* = 0.137) (Supplementary Fig. S2; Table S6). Like for fungal communities, bacterial community composition in the volatile receiving compartment was highly influenced by soil origin (Table S6, *F* = 107.4, *p* < 0.001).

When comparing the relative abundances of dominant fungal genera (relative abundance >1%) colonizing sterile soils, interesting effects of exposure to bacterial volatiles were observed. For the neutral soil (soil #2), the relative abundance of the genus *Trichoderma* in soils without exposure to bacterial volatiles was high (22.4%). Exposure to bacterial volatiles produced on REA and 1/10 TSBA plates reduced this to <2.0% (Fig. 3B). This was not seen for the acid soil (soil #1), where *Trichoderma* was by far dominant with and without exposure to bacterial community volatiles. For the alkaline soil (soil #3) a dominant fungal genus (21.5%), *Fusarium*, was reduced by exposure to bacterial volatiles produced on both REA and 1/10 TSBA plates (Fig. 3B, *F* = 48.2, *p* < 0.001), but the opposite trend was found for an unidentified genus in the class *Sordariomycota* (36.1%) (Fig. 3B, *F* = 7.804, *p* = 0.019). In soil #2, a significant increase during exposure to bacterial volatiles was seen for an unclassified genus in the family *Chaetomiaceae* (Fig. 3B, *p* = 0.031). For both soils #2 and #3, the genus *Mortierella* showed a positive response upon exposure to bacterial volatiles (Fig. 3B). Analysis of bacterial families in volatiles receiving compartments revealed a different picture. Overall, exposure to bacterial volatiles had no evident effects on bacterial family composition. The only exception was seen for soil #2 where a dominant bacterial family, *Pseudomonadaceae*, was significantly reduced by exposure to bacterial volatiles (Table S7).

### *Composition of bacteria in agar compartments and of emitted volatiles*

**Figure 4:**
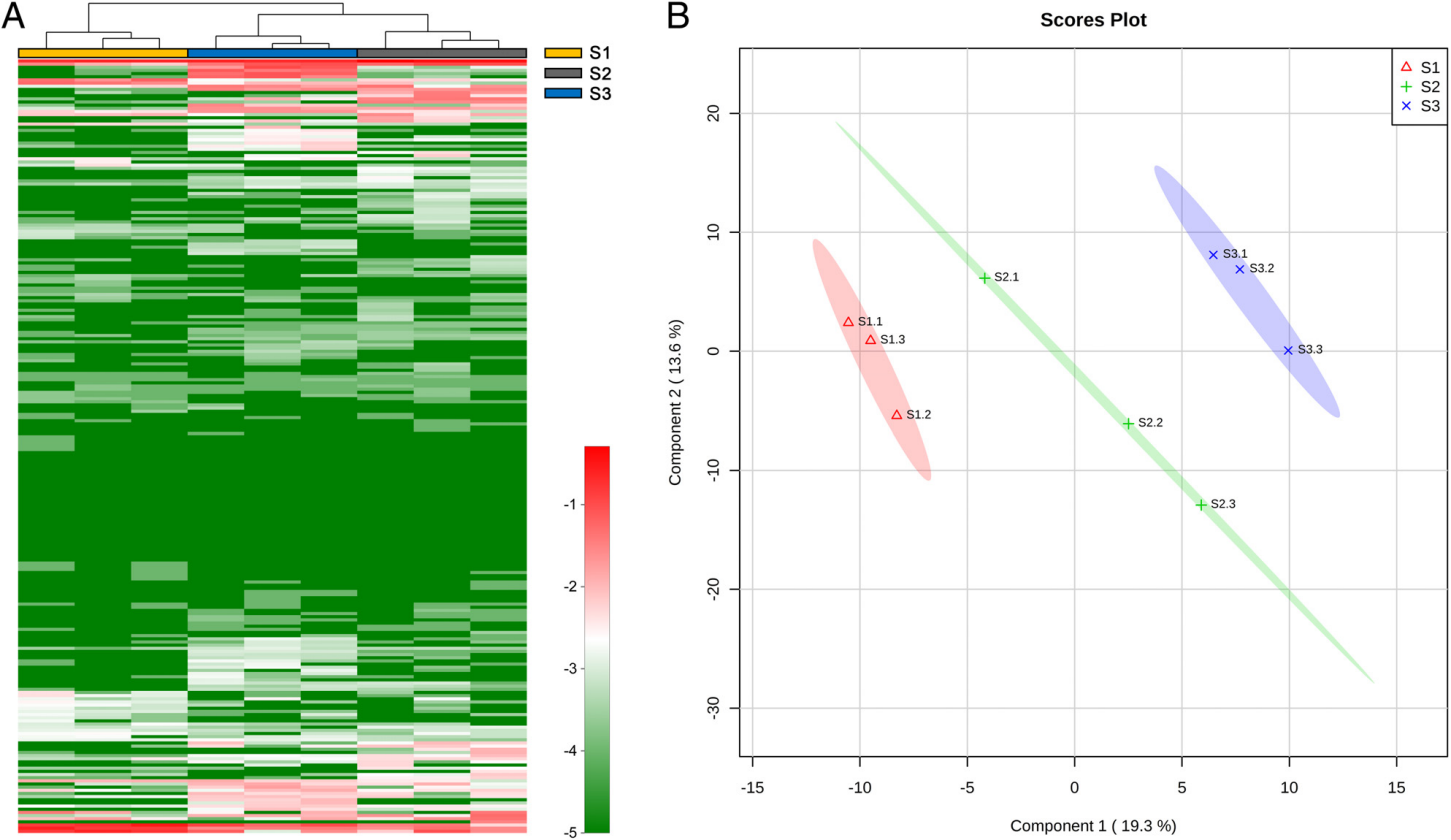
A. Heatmaps showing the composition of bacteria OTUs extracted from three agricultural soils and growing on root‐exudate agar (REA). The *x*‐axis corresponds to individual samples with three replicates per soil bacterial community. The *y*‐axis corresponds to the relative abundance of each bacterial OTU. Hierarchical cluster analysis was based on the Bray–Curtis dissimilarity with complete‐linkage method. B. Partial least square‐discriminant analysis (PLS‐DA) 2D‐score plots of volatiles profiles emitted by bacterial communities extracted from three agricultural soils (S1, S2 and S3) and growing on REA, including 95% confidence regions. The explained variances are shown in brackets.

A total of 245 bacterial OTUs affiliated with six bacterial phyla were found to be present in the volatile‐producing compartment on agar (REA and 1/10 TSBA) inoculated with filtered suspensions of the three soils. Distribution of OTUs among all agar plates revealed a core microbiome with 64% of all OTUs shared between the three soil suspensions inoculums (Fig. S3). Heatmap analysis showed that bacterial composition on both agar media (REA and 1/10 TSBA) tended to cluster for each soil origin (Fig. 4A). Soil origin had a clear impact on the volatiles blends produced by bacterial communities on REA (Fig. 4B), coinciding with different bacterial composition on these agar plates. For example, acetic acid ethyl ester, hexane‐3‐methyl and a terpene‐like compound were only detected in volatile blends of the acid soil (soil #1) bacterial community (Table 1). However, some volatile compounds were emitted by all bacterial communities, most of which were sulphur‐containing compounds (Table 1).

**Table 1:** List of volatile compounds produced by bacterial communities extracted from three agricultural soils and growing on root‐exudate agar plates.

Compound name	RT	RI	Soil #1	Soil #2	Soil #3
2‐propenenitrile	1.53	558.4	X	X	/
Sulphur dioxide^&^	2.03	582.7	X	X	X
Carbon disulphide	2.50	605.4	X	X	X
Cyclopentane methyl	2.79	622.8	X	X	/
Acetic acid, ethyl ester	2.99	629.2	X	/	/
Hexane‐3‐methyl	2.86	622.7	X	/	/
Branch alkane	3.17	638.1	/	X	/
Dimethyl disulphide*^&^	5.16	734.2	X	X	X
Benzene, (1‐methylethyl)‐^&^	11.35	921	X	X	/
α‐Pinene*	11.77	931.1	/	X	/
Benzaldehyde^&^	13.01	959.7	X	X	/
Dimethyl trisulphide*^&^	13.26	965.9	X	X	X
3‐octanone	14.04	984.1	/	/	X
Benzofuran^&^	14.42	992.9	/	/	X
Heptane, 2,2,6,6‐tetramethyl‐4‐methylene	14.45	993.9	X	X	X
Unknown mix	14.46	999	/	X	/
3‐Heptene, 2,2,4,6,6‐pentamethyl‐	14.91	1004.2	/	X	/
Unknown mix	15.92	1027.4	X	X	X
Branched alkene	16.37	1037.9	/	X	/
1‐Propene, 2‐methyl‐, tetramer	17.09	1054.3	X	X	X
1‐Octyn‐3‐ol, 4‐ethyl‐	18.4	1084.5	X	X	/
Unknown mix	18.67	1090.8	/	X	/
Unknown mix	20.03	1135.2	X	X	X
2‐methyl‐2‐bornene	22.69	1021	/	X	/
Tridecane	23.09	1199.2	X	X	X
Terpene‐like	30.54	1409	X	/	/
BHT‐quinone‐methide	32.45	1469.6	/	X	/

Shown here are only those compounds that were emitted from all replicates of each soil origin but not from the controls. Compounds were identified on basis of retention index and mass spectra.

RI—Linear retention index of 30 × 0.25 (Film 0.25) DM‐5MS‐UI column Agilent; RT‐Retention time; *verified with pure compounds; & Identified as potential fungus suppressing compounds; X, identification; /, no detection.

## Discussion

Our study revealed that antagonistic activity of volatiles produced by cultivable bacterial communities originating from three agricultural soils was predominantly targeted against fungi. Effects of volatiles emitted by bacterial communities on growth of both fungi and bacteria have not been addressed before. Fungistatic rather than bacteriostatic effects of bacterial volatiles have been reported in recent pairwise interaction studies (Garbeva *et al*., 2014b; Ossowicki *et al*., 2017). On the other hand, bacteriostatic effects of bacterial volatiles have also been observed (Audrain *et al*., 2015; Raza *et al*., 2016). Yet, pairwise interaction studies on volatile effects cannot directly be extrapolated to bacterial communities, as the spectrum of produced volatiles will be different due to the high number of contributing species and the impact of interactions (de Boer, 2017; Kai *et al*., 2018). For instance, Tyc *et al*. (2017) showed that interaction between a *Burkholderia* and a *Paenibacillus* strain resulted in strong stimulation of the *Paenibacillus* strain to produce a pyrazine volatile which was not only inhibiting the growth of *Burkholderia* but also of fungi. For simple synthetic bacterial communities (five species) it was found that both interactions and shifts in community composition had a strong effect on volatiles composition (Schulz‐Bohm *et al*., 2015). In the latter study, effects on fungi were not examined but for starving bacterial strains that were exposed to these volatiles both positive and negative effects were seen. In a study of Hol *et al*. (2015), effects of bacterial diversity on volatiles production were addressed using plated dilutions of soil suspensions. This study revealed an increase of complexity of volatile blends and of fungal pathogen suppression with increasing diversity of soil bacterial communities.

In our study, we aimed to approach the natural conditions under which fungi and bacteria are confronted with volatiles in soil by exposing developing soil microbial communities to volatiles in compartments containing sterile soil. An amount of natural soil was used to inoculate the sterile soil. Hence, no pre‐cultivation was included indicating that all microbes present in the soil inoculums could be involved in the colonization of the sterile soils. For the volatile‐producing compartments, we had to eliminate fungi from soil microbial communities in order to be able to study the effect of volatiles emitted only by bacterial communities. The manipulation step included the use of filtration and fungal inhibitors. This together with the growth conditions on agar (two treatments) has reduced the number of bacterial species that were involved in the community assembly. For instance, the family *Acidobacteriaceae* was detected among the colonizing bacteria in the receiving compartments but not in the volatile production compartments. Yet, the contribution of about 250 bacterial OTUs belonging six phyla implies a high level of complexity of bacterial communities in the volatile‐producing compartments.

Volatiles emitted from all cultivable bacterial communities had a negative effect on fungal biomass production in the receiving compartments whereas bacterial numbers were not affected. The consistency of these results is in line with our hypothesis that volatile‐mediated antagonism of bacteria is mainly directed against fungi because of differences between bacterial and fungal dispersal abilities. Production of antifungal volatiles may limit the chance for fungal hyphae to invade heterogeneously distributed nutrient patches that are occupied by bacteria. This may also be important for bacteria colonizing rhizospheres, which can also be considered as heterogeneously distributed nutrient patches (van Dam *et al*., 2016).

The increase of antifungal volatiles activity with increasing soil bacterial community diversity reported by Hol *et al*. (2015) combined with the lack of antibacterial activity of volatiles produced by cultivable soil bacterial communities in the current study points at a collective volatile‐mediated activity of soil bacteria against fungi. A possible explanation for such a collective antifungal activity is that many bacterial species share interest in limiting competition with fungi and may therefore contribute to the total pool of volatiles with antifungal activity. Analysis of the composition of the volatiles emitted by the three soil bacterial communities on REA revealed several compounds that are known to have antifungal effects. Some of these compounds, like organosulfur compounds, were produced by all bacterial communities. Others, like benzaldehyde and 3‐octanone, were emitted by 1 or 2 soil bacterial communities. Similar to that reported for other studies our study revealed that the extent of inhibition of fungi by bacterial community volatiles was influenced by the growth media (Lazazzara *et al*., 2017; Zareian *et al*., 2018). The stronger inhibition by bacteria grown on TSB‐agar is likely to be due to increased production of sulphur‐containing volatiles on TSB‐agar (Garbeva *et al*., 2014a). Besides volatile organic compounds, small inorganic volatiles like hydrogen cyanide and hydrogen sulphide may have been involved in inhibition of fungi (Piechulla *et al*., 2017). However, our analysis method did not record small inorganic volatiles.

A few studies have shown that the presence of fungal inhibiting volatiles in soils is a widespread phenomenon (van Agtmaal *et al*., 2018; de Boer *et al*., 2019). Therefore, volatiles present in the soil atmosphere may have an important contribution to soil fungistasis: the commonly observed restriction in germination of spores or growth of hyphae that fungi experience when they are in contact with soil (Garbeva *et al*., 2011). Soil fungistasis was first described by Dobbs and Hinson (1953) and has been shown to occur in almost every soil albeit it at variable intensities (Garbeva *et al*., 2011). Based on our results we propose that bacteria are major contributors to VOCs‐mediated soil fungistasis.

Several studies indicate that the sensitivity of fungal species for bacterial volatiles can differ (Kai *et al*., 2009; Garbeva *et al*., 2011). The shifts in fungal community composition that we observed in the soil microbial communities exposed to bacterial volatiles can be the result of these differences in sensitivity. Indeed, the sensitivity of fungal species to volatile blends produced by different soil bacterial communities appears to differ as we found a strong negative effect on development of *Trichoderma* sp. by volatiles derived from soil #2 bacterial communities but not by volatiles from soil #1 bacterial communities. Interestingly, also volatiles emitted from different soils were shown to have different inhibitory effects on hyphal growth of plant pathogenic fungal and oomycetal species (van Agtmaal *et al*., 2018). This indicates that the composition of soil volatiles is an important selective factor for growth of fungal species. Since our study dealt with production of volatiles by bacterial communities growing under similar conditions (the agar media), we can relate the differences in sensitivity of fungal species to volatiles to differences in bacterial community composition.

In conclusion, our study showed that volatiles produced by soil bacterial communities mainly suppressed growth of fungi. We propose that many bacterial species benefit from a collective bacterial volatile‐mediated antagonism against fungi. Next to retarding the total development of fungal biomass, selective effects on growth of fungal species became also apparent. Analogies of our findings with studies on volatile‐mediated fungistasis could imply that bacteria are major contributors to soil fungistasis. Since soil fungistasis is at the basis of general disease suppression further studies on the link between soil bacterial community composition and volatile suppression of pathogenic soil fungi may offer possibilities to enhance natural disease suppression.

## Author contributions

X.G. and W.B. conceived the project and designed the experiments; X.G. and X.J. analysed the results with assistance from X.W., A.C., W.B., M.H., and P.K.G.; X.G. and W.B. wrote the first draft of the manuscript, and P.G. contributed substantially to revisions.

## Supporting information


**Appendix S1:** Supplementary material
