# Supplementary material for: Volatile‐mediated antagonism of soil bacterial communities against fungi
Source: Environ Microbiol. 2019 Nov 4;22(3):1025–35. doi: 10.1111/1462-2920.14808 (PMC7064993; doi:10.1111/1462-2920.14808)
Supplement: Supplementary file 1 — Appendix S1: Supplementary material [file EMI-22-1025-s001.docx]

**Volatile-mediated antagonism of soil bacterial communities against fungi**

Xiaogang Li^1, 2, 3^, Paolina Garbeva^2^, Xiaojiao Liu^2,5^, Paulien JA klein Gunnewiek^2^, Anna Clocchiatti^2^, Maria PJ Hundscheid^2^, Xingxiang Wang^3^, Wietse de Boer^2, 4*^

^1^Co-Innovation Center for Sustainable Forestry in Southern China, College of Biology and the Environment, Nanjing Forestry University, Nanjing 210037, China

^2^Department of Microbial Ecology, Netherlands Institute of Ecology, NIOO-KNAW, Wageningen 6708PB, The Netherlands

^3^Key Laboratory of Soil Environment and Pollution Remediation, Institute of Soil Science, Chinese Academy of Sciences, Nanjing 210008, China

^4^Soil Biology Group, Wageningen University, Wageningen 6708PB, The Netherlands

^5^College of Plant Protection, Southwest University, Chongqing 400715, China

**Corresponding authors^*^**

Prof. Wietse de Boer, Department of Microbial Ecology, Netherlands Institute of Ecology

E-mail: [W.deBoer@nioo.knaw.nl](mailto:W.deBoer@nioo.knaw.nl),

Tel: +31-317473676

**Supplementary information**

1. **Supplementary Table** **of Contents**

Table S1. Description of the sampling sites and soil physico-chemical properties.

Table S2. Composition of growth media used in this study.

Table S3. One-way ANOVA of overall effects of volatiles produced by soil bacterial communities growing on agar media on fungal development (ergosterol content) in receiver compartments (sterile soil inoculated with fresh soil).

Table S4. Extent of inhibition of fungal biomass production in the receiver compartment (sterile soil inoculated with fresh soil) by volatiles produced by soil bacterial communities growing on Root exudates agar (REA) and 1/10 Tryptic Soy Broth agar (TSBA).

Table S5. One-way ANOVA on overall effects of volatiles produced by soil bacterial communities growing on agar media on bacterial development (qPCR based numbers) in receiver compartments (sterile soil inoculated with fresh soil).

Table S6. Results of one-way PERMANOVA using Bray-Curtis similarity values showing the effects of exposure to bacterial volatiles and of soil origins on fungal and bacterial OTU composition in the volatile receiving compartments (sterile soil inoculated with fresh soil).

Table S7. Taxonomic affiliation of the most abundant bacterial families (>0.5%) in inoculated sterile soils that were exposed (VCs) to bacterial volatiles produced on two agar media (root-exudate agar, 1/10 TSBA) or to volatiles emitted from the non-inoculated growth media (CK).

1. **Supplementary Figure** **of Contents**

Figure S1. Effect of volatiles produced by soil bacterial communities growing on two agar media (1/10 TSBA and REA; see Table S2) on fungal/bacterial ratios in receiver compartments.

Figure S2. Principal component analysis based on lineage-specific, weighted UniFrac results of the composition of bacterial community in soils in the receiver compartments that were exposed to volatiles produced by soil bacterial communities growing on two agar media.

Figure S3. Ternary plot of OTUs showing the relative abundance of OTUs present on REA and 1/10 TSBA agar plates inoculated with filtered extracts from three soils (#1, #2, #3).

**Supplementary Tables**

Table S1. Description of the sampling sites and soil physico-chemical properties.

| Soils | pH (H_2_O) | SOM (%) | Total-N (%) | Total-C (%) | C/N | Ergosterol (µg/g) | Available K (mg/kg) | Available P (mg/kg) | Moisture content (g/g) | 100 % WHC (g/g) | Texture | Coordinates | |
| --- | --- | --- | --- | --- | --- | --- | --- | --- | --- | --- | --- | --- | --- |
| #1 | 5.12 | 5.81 | 0.21 | 3.14 | 16.50 | 3.50 | 59.95 | 2.69 | 0.16 | 0.35 | Sand | 52°7′73″N | 6°34′13″E |
| #2 | 6.64 | 3.86 | 0.14 | 2.31 | 14.95 | 0.96 | 34.00 | 1.40 | 0.14 | 0.29 | Sand | 51°54′36″N | 5°85′17″E |
| #3 | 7.58 | 3.71 | 0.14 | 2.08 | 14.85 | 1.32 | 223.26 | 2.62 | 0.20 | 0.37 | Sand | 51°69′80″N | 5°84′81″E |
|  | SOM, soil organic matter | | | | | | | | | | | | |

Table S2. Composition of growth media used in this study.

| Types | Composition |
| --- | --- |
| Tryptic soy broth (TSB) agar | Tryptic soy broth 30 g/L (Sigma-Aldrich, St. Louis, Missouri, USA), agar powder CMN (Boom, Meppel, Netherlands) 20 g/L |
| Potato dextrose agar (PDA) | Potato Dextrose Agar 39 g/L (Sigma-Aldrich) |
| Root-exudate agar (REA) with fungal inhibitors | KH_2_PO_4_ 1g/L, (NH_4_)_2_SO_4_ 0.1 g/L, yeast extract (Bacto Difco, Voigt Global Distribution, Lawrence, Kansas) 0.1 g/L, CMN agar (Boom) 20 g/L; pH 6.5, and 10 mL/L filtered stock root exudates' solution, 100 mg/L cycloheximide (Sigma-Aldrich), 50 mg/L thiabendazole (Sigma-Aldrich) |
| Stock solution of root exudates | Glucose 18.4 mM, fructose 18.4 mM, saccharose 9.2 mM, citric acid 4.6 mM, lacticacid 9.2 mM, succinic acid 6.9 mM, L-serine 18.4 mM, L-glutamic acid 11 mM, L-alanine 18.4 mM |
| 1/10 TSB agar with fungal inhibitors | Tryptic soy broth 3 g/L (Sigma-Aldrich),CMN agar (Boom) 20 g/L, 100 mg/L cycloheximide (Sigma-Aldrich), 50 mg/L thiabendazole (Sigma-Aldrich) |

Table S3. One-way ANOVA of overall effects of volatiles (VCs) produced by soil bacterial communities growing on two agar media on fungal development (ergosterol content) in receiver compartments (sterile soil inoculated with fresh soil).

| Soils | Effect | Df | *F* Value | *p* Value | Significant |
| --- | --- | --- | --- | --- | --- |
| #1 | RS vs. RC | 1,6 | 16.554 | 0.015 | Yes |
|  | TS vs. TC | 1,6 | 606.787 | <0.001 | Yes |
|  | VCs vs. CK | 1,12 | 21.924 | <0.001 | Yes |
| #2 | RS vs. RC | 1,6 | 20.582 | 0.011 | Yes |
|  | TS vs. TC | 1,6 | 47.578 | 0.002 | Yes |
|  | VCs vs. CK | 1,12 | 102.248 | <0.001 | Yes |
| #3 | RS vs. RC | 1,6 | 25.864 | 0.007 | Yes |
|  | TS vs. TC | 1,6 | 128.709 | <0.001 | Yes |
|  | VCs vs. CK | 1,12 | 29.816 | <0.001 | Yes |

RS: Root exudates agar inoculated with bacterial suspensions; RC: Root exudates agar only; TS: 1/10 Tryptic Soy Broth agar inoculated with bacterial suspensions; TC: 1/10 Tryptic Soy Broth agar only; VCs: All receiver compartments (sterile soil inoculated with fresh soil) with VCs exposure; CK: All receiver compartments without exposure to bacterial VCs.

Table S4. Percentage of inhibition of fungal biomass production in the receiver compartment (sterile soil inoculated with fresh soil) by volatiles (VCs) produced by soil bacterial communities growing on Root exudates agar (REA) and 1/10 Tryptic Soy Broth agar (TSBA).

| Medium | Fungal inhibition^*^ | | |
| --- | --- | --- | --- |
|  | Soil #1 | Soil #2 | Soil #3 |
| REA | 45.3%±15.7a | 34.5%±7.2a | 54.8%±1.0a |
| TSBA | 65.3%±4.1b | 41.2%±3.6a | 81.5%±5.6b |

Mean values and standard deviations are presented (*n*=3). ^*^Fungal inhibition = - (VCs bacterial communities – VCs control)/VCs control. VCs controls represent VCs emitted from growth media without bacterial inoculums. Different letters in the column indicate that statistically significant differences (*p*<0.05) between the two media.

Table S5. One-way ANOVA on overall effects of volatiles (VCs) produced by soil bacterial communities growing on two agar media on bacterial development (qPCR based biomass) in receiver compartments (sterile soil inoculated with fresh soil).

| Soils | Effect | Df | *F* Value | *p* Value | Significant |
| --- | --- | --- | --- | --- | --- |
| #1 | RS vs. RC | 1,6 | 0.17 | 0.70 | No |
|  | TS vs. TC | 1,6 | 0.00 | 0.97 | No |
|  | VCs vs. CK | 1,12 | 0.03 | 0.87 | No |
| #2 | RS vs. RC | 1,6 | 11.18 | 0.03 | Yes |
|  | TS vs. TC | 1,6 | 0.38 | 0.57 | No |
|  | VCs vs. CK | 1,12 | 3.06 | 0.10 | No |
| #3 | RS vs. RC | 1,6 | 0.02 | 0.89 | No |
|  | TS vs. TC | 1,6 | 13.36 | 0.02 | Yes |
|  | VCs vs. CK | 1,12 | 3.37 | 0.09 | No |

RS: Root exudates agar inoculated with bacterial suspensions; RC: Root exudates agar only; TS: 1/10 Tryptic Soy Broth agar inoculated with bacterial suspensions; TC: 1/10 Tryptic Soy Broth agar only; VCs: All receiver compartments (sterile soil inoculated with fresh soil) with VCs exposure; CK: All receiver compartments without exposure to bacterial VCs.

Table S6. Results of one-way PERMANOVA using Bray-Curtis similarity values showing the effects of bacterial community volatiles (VCs) and soil origins on composition of fungal and bacterial OTUs in the VCs receiving compartments (sterile soil inoculated with fresh soil).

| Data | Soils | Factors | Df | MeanSqs | *F* Value | *p* Value | Significant |
| --- | --- | --- | --- | --- | --- | --- | --- |
| Fungal community | #1 | Bacterial VCs^1^ | 1,12 | 0.098 | 2.972 | 0.02 | Yes |
|  | #2 | Bacterial VCs | 1,12 | 0.304 | 6.973 | 0.001 | Yes |
|  | #3 | Bacterial VCs | 1,12 | 0.039 | 4.096 | 0.004 | Yes |
|  | All soils | Soil origins^2^ | 2,36 | 2.327 | 81.190 | <0.001 | Yes |
| Bacterial community | #1 | Bacterial VCs | 1,12 | 0.023 | 1.878 | 0.151 | No |
|  | #2 | Bacterial VCs | 1,12 | 0.109 | 2.621 | 0.069 | No |
|  | #3 | Bacterial VCs | 1,12 | 0.035 | 1.882 | 0.137 | No |
|  | All soils | Soil origins | 2,36 | 2.810 | 107.400 | <0.001 | Yes |

^1^Bacterial VCs indicate the effect of VCs emitted by bacterial communities growing on both agar media as compared to VCs emitted by the media without bacteria, ^2^Soil origins represents the effect of soil types.

Table S7. Taxonomic affiliation of the most abundant bacterial families (>0.5%) in inoculated sterile soils that were exposed to bacterial volatiles (VCs) produced on two agar media (root-exudate agar, 1/10 TSBA) or to the VCs emitted from non-inoculated growth media (CK).

| Family | Soil #1 | | | | Soil #2 | | | | Soil #3 | | | |
| --- | --- | --- | --- | --- | --- | --- | --- | --- | --- | --- | --- | --- |
|  | REA | | TSBA | | REA | | TSBA | | REA | | TSBA | |
|  | CK | VCs | CK | VCs | CK | VCs | CK | VCs | CK | VCs | CK | VCs |
| Pseudomonadaceae | 0.75% | 0.54% | 0.58% | 0.76% | 29.38% | 9.82% | 14.68% | 5.83% | 55.06% | 52.35% | 60.46% | 50.79% |
| Unclassified | 24.21% | 33.28% | 31.54% | 32.61% | 11.20% | 12.74% | 5.94% | 10.19% | 15.65% | 13.66% | 9.55% | 5.33% |
| Chitinophagaceae | 2.64% | 2.07% | 1.89% | 1.92% | 26.11% | 58.53% | 53.57% | 55.58% | 0.71% | 0.52% | 0.74% | 1.89% |
| Xanthomonadaceae | 26.01% | 21.65% | 25.87% | 20.27% | 1.04% | 1.02% | 0.66% | 0.80% | 0.88% | 1.16% | 0.93% | 1.07% |
| Burkholderiaceae | 12.63% | 11.54% | 12.92% | 14.97% | 14.50% | 5.69% | 11.97% | 7.45% | 0.04% | 0.03% | 0.03% | 0.01% |
| Streptomycetaceae | 0.57% | 0.48% | 0.44% | 1.02% | 0.15% | 0.35% | 0.14% | 0.54% | 6.02% | 7.04% | 4.17% | 9.41% |
| Sphingobacteriaceae | 3.52% | 5.58% | 4.29% | 6.06% | 0.30% | 0.27% | 0.17% | 0.39% | 0.36% | 2.72% | 0.69% | 4.26% |
| Acidobacteriaceae Subgroup_1 | 6.10% | 4.49% | 5.28% | 5.45% | 0.21% | 0.15% | 0.17% | 0.17% | 0.04% | 0.06% | 0.03% | 0.04% |
| Comamonadaceae | 0.43% | 0.66% | 0.32% | 0.38% | 1.44% | 1.35% | 1.14% | 4.25% | 2.10% | 2.05% | 2.66% | 3.61% |
| Micrococcaceae | 0.37% | 0.66% | 0.40% | 0.63% | 0.96% | 0.68% | 0.61% | 1.62% | 2.97% | 2.42% | 3.34% | 3.62% |
| Bacillaceae | 3.04% | 2.61% | 2.04% | 2.32% | 0.53% | 0.23% | 0.33% | 0.28% | 0.55% | 0.44% | 0.69% | 0.57% |
| Sphingomonadaceae | 1.70% | 1.63% | 1.39% | 1.03% | 1.02% | 0.58% | 0.44% | 0.47% | 1.24% | 0.82% | 1.14% | 1.20% |
| Rhizobiaceae | 0.72% | 0.75% | 0.50% | 0.43% | 1.23% | 0.56% | 0.61% | 0.54% | 1.64% | 1.21% | 2.08% | 0.94% |
| Oxalobacteraceae | 0.29% | 0.28% | 0.25% | 0.26% | 1.34% | 0.49% | 2.10% | 1.15% | 0.30% | 3.05% | 0.51% | 0.85% |
| Bradyrhizobiaceae | 2.04% | 1.53% | 1.21% | 1.31% | 0.78% | 0.39% | 0.40% | 0.64% | 0.51% | 0.30% | 0.54% | 0.47% |
| Flavobacteriaceae | 0.04% | 0.04% | 0.02% | 0.02% | 1.99% | 2.51% | 1.60% | 2.52% | 0.05% | 0.05% | 0.05% | 0.17% |
| Rhizobiales_Incertae_Sedis | 1.72% | 1.22% | 1.51% | 1.46% | 0.18% | 0.08% | 0.11% | 0.12% | 0.06% | 0.08% | 0.06% | 0.04% |
| Cytophagaceae | 0.03% | 0.02% | 0.01% | 0.02% | 0.71% | 0.60% | 0.44% | 1.44% | 0.50% | 0.39% | 0.67% | 1.40% |

“REA”: root-exudate agar, and “TSBA”: 1/10 tryptic soy broth agar. CK: the control of corresponding media without bacterial communities; VOCs: the treatment of corresponding media with soil bacterial communities.

**Supplementary Figures**


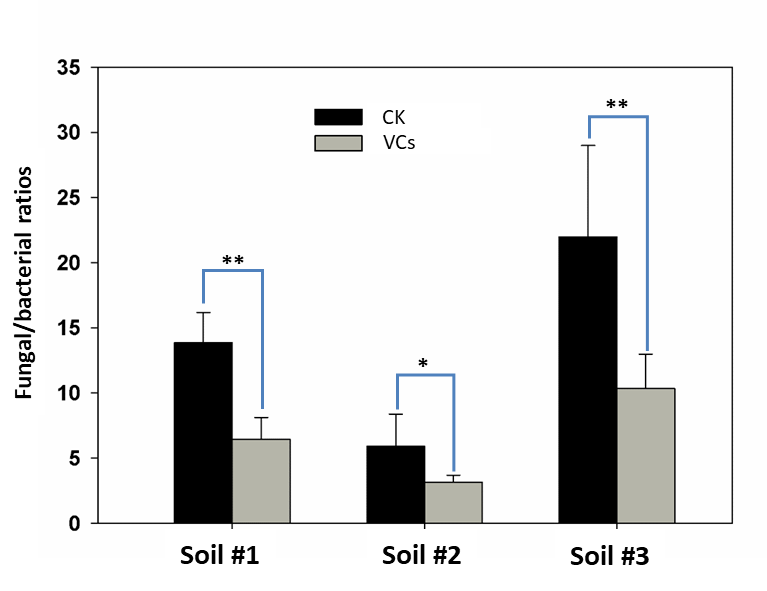


Figure S1. Effect of VCs produced by soil bacterial communities growing on agar media on fungal/bacterial biomass ratios in receiver compartments. Mean values and standard deviations are presented (*n*=6). Asterisks indicate that differences between soils with (VCs) and without (CK) exposure to bacterial VCs are statistically significant (* *p* < 0.05, ** *p* < 0.01) as determined by Fisher’s LSD test.


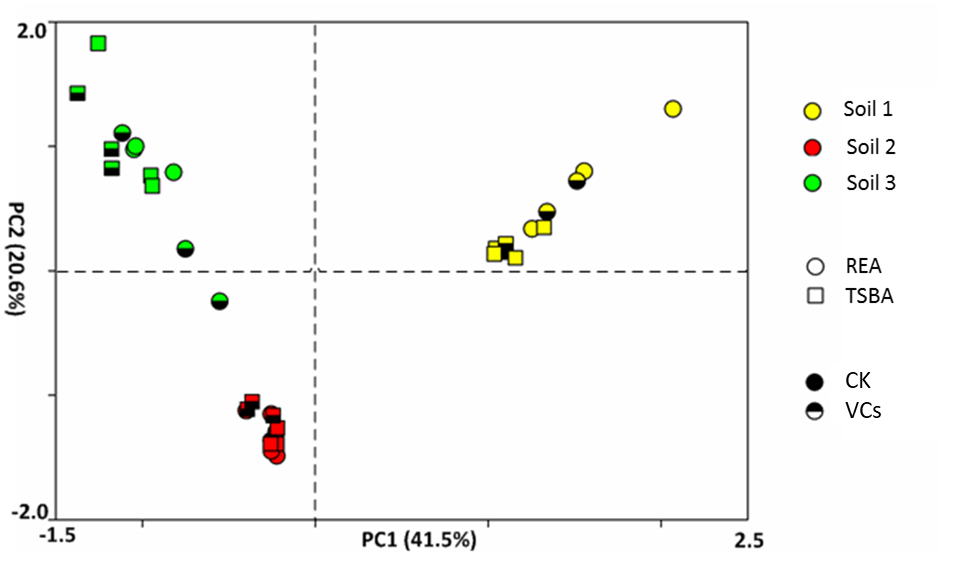


Figure S2. Principal component analysis based on lineage-specific weighted UniFrac results of the composition of bacterial community in soils in the receiver compartments that were exposed to (VCs) or not (CK) bacterial volatiles produced by soil bacterial communities growing on two agar media. “REA”: root-exudate agar, and “TSBA”: 1/10 tryptic soy broth agar.


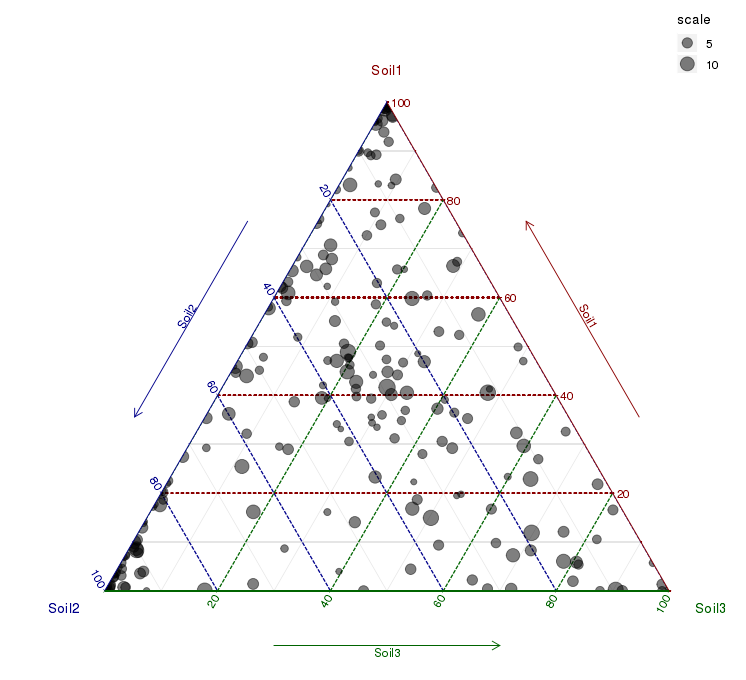


Figure S3. Ternary plot of OTUs showing the relative abundance of bacterial OTUs present on REA and 1/10 TSBA agar plates inoculated with filtered extracts from three soils (#1, #2, #3). The ternary plot for bacterial composition among the three soils was created with ggplot2 in R. Generally, the center of the ternary plot shows the core microbiome across the three soils. The OTUs uniquely associated with a specific soil corresponded to the points within the corners of the ternary plot. Diameter of plotted points corresponds to relative abundance of the OTUs.
